# Annotation of Trauma-related Linguistic Features in Psychiatric Electronic Health Records for Machine Learning Applications

**DOI:** 10.21203/rs.3.rs-2711718/v1

**Published:** 2023-03-28

**Authors:** Eben Holderness, Bruce Atwood, Marc Verhagen, Ann Shinn, Philip Cawkwell, James Pustejovsky, Mei-Hua Hall

**Affiliations:** Brandeis University; McLean Hospital; Brandeis University; McLean Hospital; Bay Area Clinical Associates; Brandeis University; Harvard Medical School

## Abstract

Psychiatric electronic health records (EHRs) present a distinctive challenge in the domain of ML owing to their unstructured nature, with a high degree of complexity and variability. This study aimed to identify a cohort of patients with diagnoses of a psychotic disorder and posttraumatic stress disorder (PTSD), develop clinically-informed guidelines for annotating these health records for instances of traumatic events to create a gold standard publicly available dataset, and demonstrate that the data gathered using this annotation scheme is suitable for training a machine learning (ML) model to identify these indicators of trauma in unseen health records. We created a representative corpus of 101 EHRs (222,033 tokens) from a centralized database and a detailed annotation scheme for annotating information relevant to traumatic events in the clinical narratives. A team of clinical experts annotated the dataset and updated the annotation guidelines in collaboration with computational linguistic specialists. Inter-annotator agreement was high (0.688 for span tags, 0.589 for relations, and 0.874 for tag attributes). We characterize the major points relating to the annotation process of psychiatric EHRs. Additionally, high-performing baseline span labeling and relation extraction ML models were developed to demonstrate practical viability of the gold standard corpus for ML applications.

## Background And Significance

Psychotic and mood disorders are among the most disabling conditions worldwide[1–5], yet there is considerable clinical heterogeneity among patients. As a result, effective personalized treatments require a better understanding of this broad diversity in patient clinical histories. Among the different factors contributing to this heterogeneity, childhood trauma stands out as an under-recognized source and a major risk for the development of severe psychiatric disorders.[6–11] Among individuals with severe psychiatric disorders, the prevalence of childhood sexual, physical, and emotional abuse is significant at 26%, 39% and 34%, respectively,[12] and the average rate of all forms of trauma in adult females is 69% and in males 59%.[10] Substantial evidence indicates that individuals exposed to childhood physical or sexual abuse are 2–3 times more likely to develop a psychiatric disorder later in life, including psychosis. [6, 13–16] Research shows that childhood traumas impact critical windows of brain development and can trigger the onset of psychosis and other psychiatric disorders. In addition, among patients with psychotic and mood disorders, childhood trauma influences psychopathology, leading to more severe symptoms, higher rate of relapses or rehospitalization, higher rates of substance abuse, higher rates of treatment resistance, and poorer functioning.[11–13, 17–20] Although evidence clearly indicates that childhood trauma contributes to psychiatric risk and poor treatment outcome, large-scale computational approaches to stratify subpopulations, extract trauma features (e.g., frequency, type), and examine the links or the impact of trauma features on psychopathology and treatment outcome have yet to be developed.

The solutions to this major gap require leveraging computational methods over large data, human expertise, and the transdiagnostic approach (Research Domain Criteria [RDoC] framework operating on dimensional conceptualizations of mental illnesses) to provide clinical insights and utilize large data for accurate patient stratification. Such explainable machine learning (ML)[21] knowledge is the key to guide development of new therapeutic targets or interventions. Electronic health records (EHRs) contain structured and unstructured data over a patient’s treatment history. Unstructured data are richly detailed and add valuable contextual information but require additional processing to extract data elements suitable for aggregate analyses. Manual chart review[22, 23] is an impractical approach and is not cost-effective. Natural language processing (NLP) tools using EHRs have been developed,[24, 25] which mine EHR data in a manner that is not only cost efficient, scalable and systematic, but could improve clinical precision by accurately classifying or stratifying patient subpopulations. For these tools to work effectively over EHR data, however, there needs to be an initial recognition of what textual aspects of the EHR help in classifying these populations. This is accomplished through annotation.

The goal of the annotation is to mark up (tag) clinical information present in the EHRs (e.g., events, symptoms, temporal expressions, etc.) in order to train NLP models to automatically extract this information from clinical text. The Informatics for Integrating Biology and the Bedside (i2b2) project is one example of a major effort which has successfully developed annotation guidelines, gold standard datasets, and resources for use by the medical community.[26–29] However, i2b2 focuses on medical related conditions (e.g., diabetes) and concepts. To our knowledge, there is currently no “computational version of chart reviews” tool that systematically extracts trauma related features and stratifies patients into subgroups (with or without childhood trauma history). In addition, annotation guidelines representing psychiatric patients and trauma-related features have yet to be developed. A gold standard corpus (i.e., annotated EHR text dataset) specifically tailored to identify trauma-related events, risk factors, symptoms, and relations between them does not exist. The lack of such resources is a major barrier in the progress for the development of accurate ML classifiers and prediction models in psychiatry.

The contributions of this study are to: 1) develop an annotation scheme and clinically informed guidelines for labeling trauma related linguistic features; 2) create training and testing corpora of psychiatric patient EHRs and establish a publicly available gold standard dataset, “Trauma Enriched Psychiatric Corpus” (TEPC) and 3) use the TEPC to train and evaluate ML models. To our knowledge, the set of resources developed in this study is the first to detect/identify linguistic features of trauma related concepts in patients with psychosis and mood disorders.

## Materials And Methods

### Dataset

EHRs were sourced from the Research Patient Data Registry (RPDR),[30] a centralized regional data repository which contains discharge summaries, encounter notes, and visit notes for over 7 million patients across all institutions in the Mass General Brigham healthcare network from 1999 onwards. Using the RPDR query tool, we aggregated all EHRs of patients who met three criteria:

1. Diagnoses of both PTSD and a psychotic disorder (schizophrenia, bipolar, schizotypal, delusional, or other non-mood psychotic disorder) at some point in patient history.
2. Between 18 and 44 years of age at time of query (Oct. 2021).
3. At least one note related to psychotherapy, symptoms referent to mental disorders, or social counseling.

**Figure 1:**
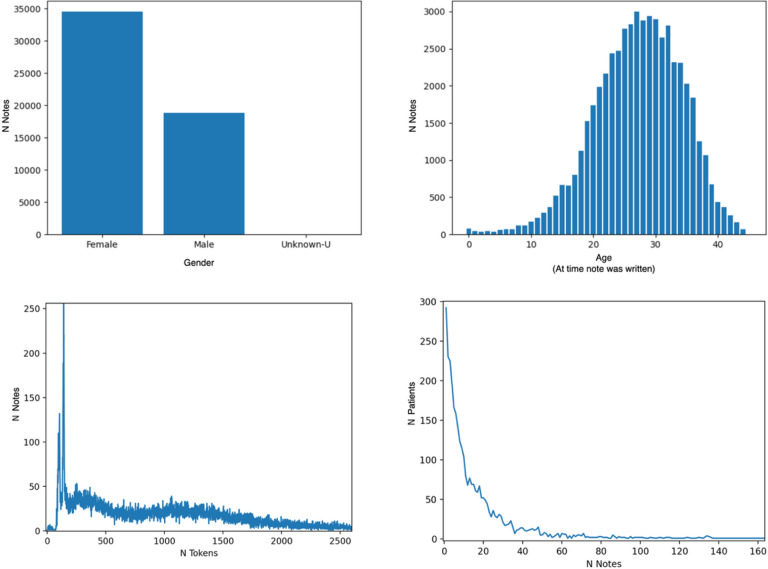
Per-note demographic and structural characteristics of the RPDR cohort. Clockwise from left: gender breakdown, number of notes by age at the time note was written, number of tokens per note, and number of notes per patient. Note that when selecting notes for annotation, we only chose from notes that were written when the patient was over 18.

This search returned EHRs for 3,515 patients, totaling approximately 55,000 notes. Data statistics including demographic information, notes per patient and tokens per note are shown in Fig. 1.

A total of 101 clinical notes were annotated to create the gold standard corpus. The dataset was constructed to be highly generalizable across age and gender, with a dense concentration of annotatable content. This was accomplished by conducting a “regular expression” search (detailed below) on the results of the RPDR query, counting instances of traumatic event keywords. The notes were first sorted by age and gender, with the most event-rich notes taken in equal portions from each demographic bucket.

### De-identification and pre-annotation processing

The 101 clinical notes were first de-identified of any individually identifiable information per HIPAA guidelines. The documents were manually reviewed, removing any of the 18 types of identifiers that are listed under the HIPAA Privacy Rule Safe Harbor method < https://www.hhs.gov/hipaa/for-professionals/privacy/special-topics/de-identification/index.html>. Names were mapped to unique labels to preserve the differentiation of referents, i.e., John Doe → <patient>, Dr. Alice Bob → Dr. <person1>, etc.

Prior to manual annotation, the documents were automatically annotated with explicit instances of trauma-related symptoms, events, and substance abuse, which were adapted from Jackson et al.[31] and are provided in the **Pre-annotation** section of the supplementary materials. Automatic pre-annotation was employed to reduce annotator burden and to ensure high recall in the annotation process by systematically marking all trivial instances of tag classes. The result of this process would then pass to the manual annotation stage to mark more complex cases.

### Annotation scheme and guidelines

**Figure 2:**
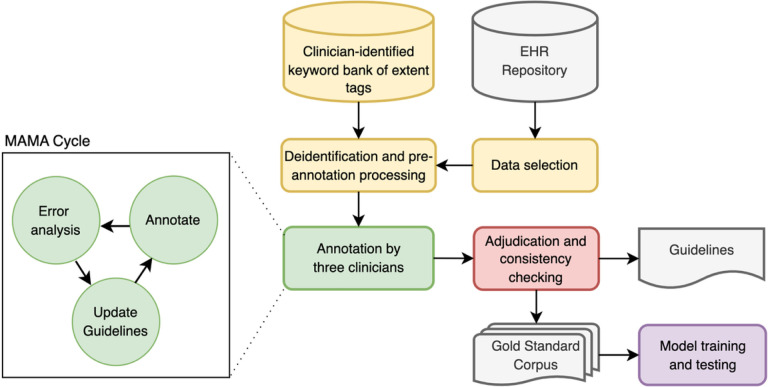
Graphical description of the project workflow. The yellow elements denote the dataset creation and preprocessing steps; green elements denote the annotation process; red denotes the revision stage; gray elements denote the inputs and outputs including the resulting Gold Standard Trauma Enriched Psychiatric Corpus (TEPC); purple refers to the baseline models trained using the Gold Standard TEPC.

The development of annotation guidelines followed the Model-Annotate-Model-Annotate (MAMA) cycle, [32] depicted in Fig. 2. Draft annotation guidelines were developed early in the project using an initial set of annotation span tag categories, relations between span tags, and attributes for both that were deemed clinically relevant. These were used to conduct a pilot annotation, followed by discussion and guideline updates.

**Figure 3:**
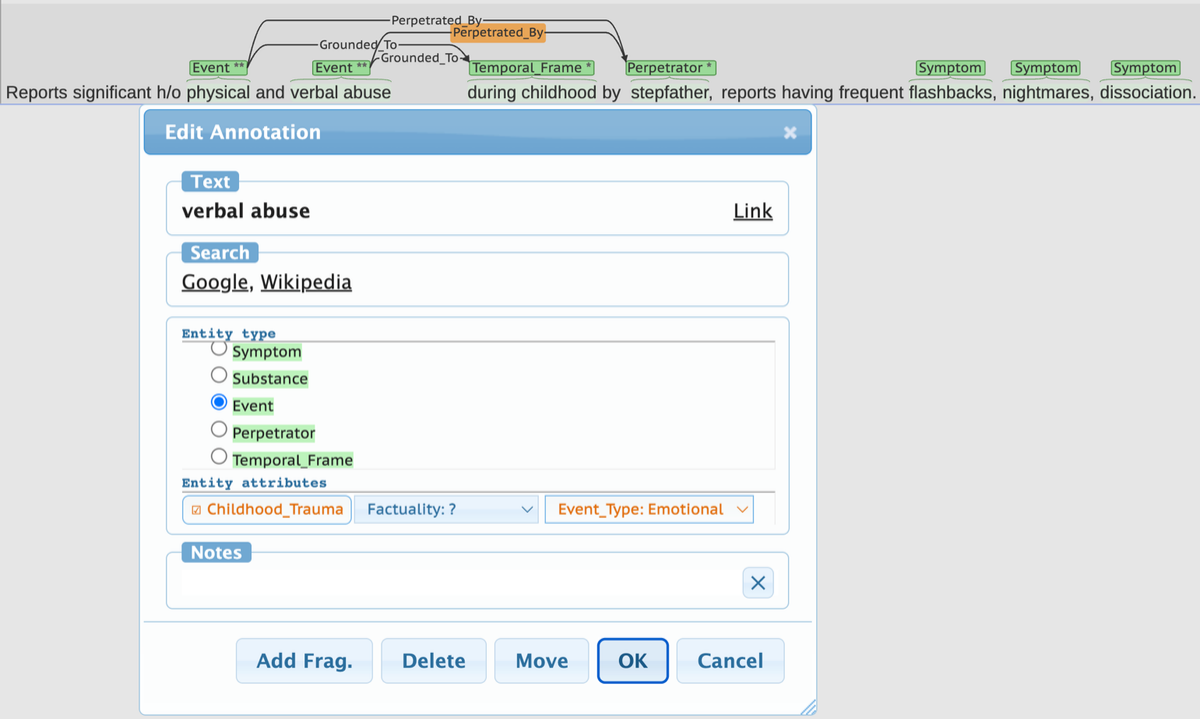
A screenshot of the BRAT annotation interface. When highlighting a span of text or drawing a relation between two annotated spans, a popup window is shown to the annotators where they may select the appropriate tag.

Annotations were completed by a team of three domain experts (MH, AS, PC) using the BRAT rapid annotation interface,[33] an open-source natural language annotation tool. Annotators first manually validated the automatically generated annotations. Relation tags were then created between pertinent span tags and assigned attribute labels to both the span tags and relations. An examples is given in Figures 3.

The guidelines were iteratively refined by the domain experts in consultation with the team of NLP researchers, using a consensus-based approach, broadly following a set of guiding principles:

1. Annotators should approach the text with a neutral stance. They should not make inferences regarding psychiatric diagnoses, nor make assumptions that are not plainly made by the text itself.
2. Annotations should maximize the pertinent clinical information.
3. Annotations should optimize model performance by maximizing replicability (reducing inter-rater disagreement) and minimizing the complexity of the machine learning task.

### Error analysis

Though the second and third principles are generally in contention, where an increase in clinical complexity leads to a more difficult modeling task, maximizing replicability is invariably beneficial. The primary concern in developing an annotation guideline is consistency; the performance of any model depends foremost on a congruent set of patterns from which it may draw inferences. To this end, annotation discrepancies were analyzed and corrected at two levels: the intra-document scope through dual annotation (local error analysis) and at the inter-document scope through tag context (global error analysis).

### Local error analysis: mini batch dual annotation

At several points during the MAMA cycle, small batches of notes (23 notes in total) were annotated by two independent annotators rather than one. These 23 dually annotated notes allowed for a direct comparison between annotators, who then discussed disagreements with the goal of harmonizing annotation and fine-tuning the annotation guidelines.

Inter-Annotator Agreement (IAA) scores were calculated over these 23 dually annotated documents using the pairwise F1 metric (defined as the harmonic mean of precision and recall), which were then averaged across each annotator pair to get a single score for each span tag, relation, and attribute tag. For relation tags, we compute both a standard F1 and a ‘relaxed’ F1. The relaxed F1 score only takes into account the relation tags whose span tag constituents are in exact agreement between annotators (a total of 255 relation annotations being compared). By comparison, the standard F1 metric takes into account all relation tags (403 relation annotations), including those whose span tag constituents are not in agreement and therefore treated as being in disagreement by default.

### Global error analysis: left context of spans

Error analysis performed during dual annotation will find only local inconsistencies, i.e., differences in how annotators approach a specific text span, not whether the approach is consistent across documents. Therefore it is also necessary to check for inconsistency at the global scope. This process focused particularly on the variation of tag extents, as extent is not as dependent on context as other features such as attributes.

To identify global inconsistencies, we developed a tool to analyze the left context of all annotated spans. First, we calculated the pointwise mutual information (PMI) between each tagged span and the token to its immediate left using the standard definition of PMI:

$$PMI(x,y)=\text{log}\left(\frac{P(xy)}{P(x)P(y)}\right)$$

Where × is the token to the left, y the span, P(xy) the expected probability of the two occurring together, P(x) the probability of the token to left occurring and P(y) the probability of the span.

**Figure 4:**
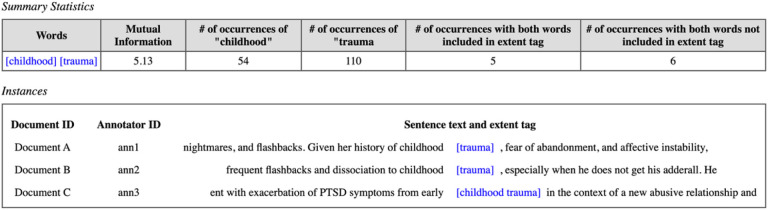
Analysis of annotations including the span “trauma” and preceding token “childhood.” A high mutual information score of 5.13 indicates potentially sufficient support of the combined annotation “childhood trauma” for use in annotation and modeling. In this example, across three documents A, B, and C one annotator annotates all of “childhood trauma”, while two other annotators only include “trauma.” These differences would not be found by comparing dually annotated notes as these notes were not dually annotated.

If the mutual information is high (> 5) and the span and the token were annotated together somewhere in the corpus, we discussed which annotation extent was preferable and revised the guidelines accordingly. An example of the interface is presented in Fig. 4.

### Adjudication and Consistency Checking

When a consensus was reached regarding all conflicts and the annotation guidelines were finalized, the Gold Standard Corpus, TEPC, was considered complete. The dually annotated notes were adjudicated, resolving all discrepancies between individual annotators, and all other notes were updated to reflect the current version of the guidelines.

### Baseline models

**Figure 5:**
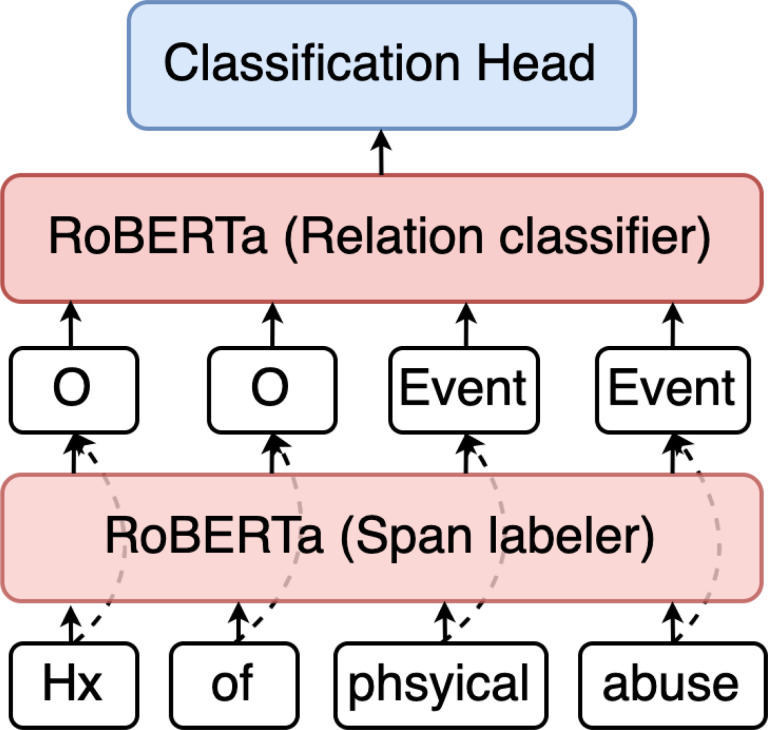
Baseline model.

Our baseline models, pictured in Fig. 5, were trained on the gold standard TEPC to validate that the annotations are learnable and to serve as a foundation for future machine learning work. Modeling consisted of two components: a span-level tagger that labels each token in the input text and a classifier that takes each valid pair of tags from the span-level tagger and labels the relationship between them.

The span-level tagger is conceptually similar to named entity recognition (NER), where the problem is approached as a sequence-to-sequence token classification task. A RoBERTa-Base[34] transformer block takes a pre-tokenized sentence as input and labels each token either as ‘O’ (signifying no span label) or as one of the span tags from the annotation specification.

The relation classifier also uses the RoBERTa-Base transformer block as its primary component but is structured as a single-label sentence-pair classification task. To generate the training data for this model, we first generate all possible relations between the span tags in a sentence and then mark the pairs that exist in the gold standard with their respective label. All other pairs are treated as examples of non-relations.

For both models, we employed a hyperparameter sweep using a Bayesian search method to find values for learning rate, batch size, number of training epochs that optimized the F1 score of the model when evaluated on the test set. Because the Sub-Event tag occurred with such low frequency in the dataset, we excluded it when developing our baseline models as there was not sufficient data to train on. Further details regarding model configuration and hyperparameter settings are found in the supplementary materials section **Baseline model configuration and hyperparameters.**

## Results

### Annotation guidelines (TraumaML)

TraumaML is designed for annotation of EHRs for patients with diagnosis of PTSD and psychotic and mood disorders. Detailed annotation guidelines are presented in the **Annotation guidelines** section of the supplementary materials.

The gold-standard guideline contains five span tags and three relation tags. Below are the five span tags that mark spans of text and the three relation tags that connect span tags.

Span Tags:

1. **Event:** The traumatic event that a patient has experienced.
2. **Perpetrator:** The perpetrator of the traumatic event.
3. **Symptom:** Symptom exhibited by the patient.
4. **Substance:** Any text span that gives information about a substance use disorder.
5. **Temporal_Frame:** A temporal grounding for the traumatic event.

Relation Tags:

- **Perpetrated_By:** Connects an event to a perpetrator.
- **Grounded_To:** Connects an event to a temporal frame.
- **Sub-Event:** Relation between an event and another event that it is part of.

For **Event** span tags, we focused on three types of traumatic events that are interpersonal in nature (i.e., sexual, physical, and emotional abuse). Trauma events that were witnessed but not directly experienced by the patient (e.g., witnessing the death of a loved one) were categorized as “other.” Events that could be perceived as traumatic but that were not forms of interpersonal trauma (e.g., physical or psychological injury from a natural disaster or motor-vehicle accident) were marked as “other”. Trauma-related terms that reflect a medical condition (e.g., traumatic brain injury) were not annotated. (See the final guidelines for details.)

For **Symptom** span tags, we annotated all symptoms, not just those that were documented by clinicians as being associated with specific trauma events. This is because trauma is associated with a wide range of psychopathology (psychosis, mood, anxiety), not only to posttraumatic stress symptoms (e.g., flashbacks, nightmares), and because symptoms may overlap across diagnostic conditions (e.g., sleep disturbance is seen across multiple psychopathologies).

**Substance** span tags were annotated only if they reflected clinically defined substance abuse/dependence such as “alcohol use disorder,” or employed homologous language such as “complicated withdrawal,” “detox,” or “daily heroin use.” Text not directly indicative of abuse/dependence, such as positive results of toxicology screens or other unspecified drug/alcohol use, was not considered, and no differentiation between current and past substance abuse was made.

The earliest versions of the guidelines were iteratively modified on the basis of the feedback from annotators. Major modifications included the following:

- Partial removal of the relation tag **Grounded_To** that was originally intended to also link symptoms to a temporal frame. In the final version of the guidelines **Grounded_To** only links to **Event** spans.
- Removal of the relation tag **Symptom_Associated_With** that was originally intended to link symptoms to trauma events. The rationale for this change is that with the exception of posttraumatic stress symptoms in which a patient has intrusive re-experiencing symptoms (e.g., nightmares, flashbacks, etc.) after a traumatic event, it is difficult to establish clear relations between symptoms and traumatic events.
- Removal of the relation tag **Equal_To** that was intended to indicate that two or more trauma **Event** tags referred to the same event. Instead, we asked annotators to focus on **Sub-Event**, in which an **Event** is followed by an elaborating text such as “physical abuse by father hitting patient with an object”.
- Removal of the **Family_Environment** span tag because it was difficult to reach a consensus definition about what types of spans to include in this span tag.

### Gold-standard “Trauma Enriched Psychiatric Corpus” (TEPC)

Table 1 shows the distribution of the gold standard TEPC. Overall, TEPC contains 7,988 span tags and 680 relation tags. Most frequent span tags are **Symptom** (6,022), followed by **Event** (800), then **Substance** (604). Inter-annotator agreement results from the 23 dually annotated documents are reported in Table 2.

### Model performance

Per-label results for the baseline model setup are reported in Table 3, using the optimal hyperparameters that were identified through the hyperparameter sweeping described in the Methods section. Results for the span-level model and relation classification model are independent – we do not use the outputs of the span-level model as the inputs to our relation classifier, and instead use the gold standard spans to evaluate relation classification performance. This allows for a measurement of the model in ideal conditions and avoids issues of error propagation that are inherent in pipelines involving multiple models. The span-level model achieved a macro-F1 of 0.49 and the relation extraction model achieved a macro-F1 of 0.71.

## Discussion

To the best of our knowledge, this study represents the first investigation of trauma-related features in a large and representative cohort of psychiatric electronic health records. In this study we developed clinically informed guidelines for annotating these patient health records for instances of traumatic events, created a gold standard publicly available dataset, and demonstrated that the data gathered using this annotation scheme is suitable for training a ML model to identify indicators of trauma in novel (unseen) health records. The preprocessing steps, guidelines, as well as the code for NLP models, are available as a repository on our GitHub group < https://github.com/TraumaML/JAMIA-Materials/>.

We identified five span tags (**Event, Perpetrator, Symptom, Substance**, and **Temporal_Frame**) and three relational tags (**Perpetrated_By, Grounded_To**, and **Sub-Event**) as important for modeling trauma-related linguistic features in psychiatric EHR data. Sub-Event tags were not included in baseline modeling due to low support and limited agreement among annotators.

Identifying symptoms and trauma-related features in the mental health domain is challenging because psychiatric clinical narratives have considerable variability, both because of the heterogeneity of patient presentations as well as heterogeneity in the way that different clinicians may document clinical events.

Nuance is intrinsic to the annotation process of psychiatric EHRs, and in many cases there is no obvious justification for a correct way to annotate a span. The following discussion aims to shed light on the consistent points of disagreement and complexity in the annotation process and how we navigated these challenges. Our efforts serve as a guide for future annotation endeavors, providing insight into how to effectively identify symptoms and trauma-related features while accounting for the inherent nuance and variability of clinical narratives.

To guide our annotation process, we therefore relied on three overarching principles: (1) to approach the text neutrally; (2) to maximize the pertinent clinical information; and (3) to optimize model performance by minimizing complexity. Principles #2 and #3 are generally in contention; any increase in annotation complexity (more span tags, attributes, and relations, or an expanded reading frame) comes at the cost of a more difficult learning task with fewer training instances of increasingly specific descriptions. The guidelines were developed based on the project scope and the capabilities of the model, in order to make meaningful conclusions using the fixed training corpus size. To illustrate how these principles were substantiated in the guidelines, we include the resolutions to several major areas of disagreement during the annotation process.

In some cases, annotators found it difficult to decide the truthfulness/credibility of a traumatic event, due to the acute state of psychosis of a patient (e.g., delusional). In these instances, events were assigned the default attribute, *factual*. Annotators marked attributes *maybe* or *unlikely* only when the sentential context is unambiguous that the event in question is uncertain or dubious. Thus, “sexual assault” in the span “possible sexual assault” was attributed as *maybe*, but in the line “pt noted that she had been assaulted…” further in the same document, the span “assaulted” was attributed as *factual*. This conservative approach is an exemplification of principle #1, where the text is taken at face value based on the current limitations of language models to infer nuanced context, especially over prolonged reading frames. Substance use was similarly annotated, where unspecified drug/alcohol use was ignored when the clinician did not specify it as a clinical problem.

Additionally, many questionable situations during annotation required balancing annotation complexity with modeling complexity. For instance, to establish consistency on span tag extents, the extent of a span tag was determined as the minimal extent to capture the information pertinent to the study. An example of this would be deciding to annotate “self-injury” as a symptom only, rather than the full span “scratch herself superficially as self-injury.” Although this decision results in the loss of clinical context, the inclusion of additional information would be deemed extraneous if the training data does not provide sufficient support for a model to draw an inference about the symptom in question. In this way principles #2 and #3 are balanced. Importantly, this balance is contingent on the scope of the study. Because we were focused specifically on childhood trauma, we decided to only annotate “trauma” within the context “military trauma”, as the gains in specificity do not offset the loss in generalizability.

To simplify the annotation task and still capture specific temporal links between temporal expressions such as dates, and available events ({sexual abuse} in {2002}), we created the **Temporal_Frame** tag, consisting of 5 possible temporal type attributes: age, duration, time-of-life, major event, and date. The relation between the temporal frame and the event is expressed with the **Grounded_to** relation. We do not include other temporal relations such as defined by the Temporal Relation (TLINK) link described in the NIMH-THYME (Temporal History of Your Medical Events) guidelines as it is beyond the project scope. Although a framework of temporal relations to the onset of psychosis symptoms has been reported,[35] this framework focuses on psychosis symptom onset identification and is based on a limited set of symptom keywords which are not suitable to capture more complex linguistic variants. Developing temporal NLP systems in mental health records remains a challenge, due to the inherent complexity of the task. We plan to develop a psychiatric temporal relation annotation scheme and build temporal information extraction systems for psychiatric notes using a graph neural network method in the future.

### Model error analysis

The performance of the baseline models demonstrates the practical viability of the gold standard TEPC for machine learning applications. Incorrect predictions made by the baseline models primarily fall into one of three categories: error in predicting span tag extents, error in classifying relations, and difficulties distinguishing between syntactic and semantic validity. For example, in the line “mother offered support to patient,” the span “mother” was incorrectly annotated as a perpetrator. To address these issues, we will finetune the transformer blocks in the models on the entirety of the psychiatric clinical narratives in the RPDR, redesigning our model architecture to a joint modeling approach that predicts both the text spans and their relations in a single step, and modifying the model’s input representation to include temporally preceding information in a document.

In comparing the performance of the baseline models to the clinical experts involved in the annotation task, it is challenging to establish a direct comparison for the relation extraction model. This is because when providing the inputs to the relation extraction model, we provide the span tags from the TEPC, where span-level disagreements have been resolved during adjudication. Therefore, errors introduced by span-level disagreement (e.g. one annotator marking < shoved > and < father > with a **Perpetrated_By** relation between them, and another annotator marking < shoved > and < his father > with a **Perpetrated_By** relation between them) are not present in the model evaluation but are present in the human annotator error rate evaluation. As a result, the reported performance of the relation model is expectedly higher than the reported human performance, despite human performance generally being considered an upper bound of potential accuracy on the task. Computing the relaxed F1 metric ameliorates the issue of span-level disagreement but also significantly reduces the number of instances used to compute the human baseline, as any relation tags with span-level disagreements are excluded.

### Limitations and related future considerations

There are a few limitations that should be highlighted. First, given the complexity of psychiatric clinical notes and expertise required of annotators, the annotated corpus size is small relative to other domains (e.g., recipes). This is a major limiting factor for training ML models. We are developing new methodologies to scale up the amount of de-identified and annotated data in the corpus. Second, among the annotated corpus, 23 notes were annotated by two annotators whereas the remaining 78 were not. However, these 78 singly annotated notes were retroactively updated to reflect changes to the guidelines that occurred while resolving discrepancies in the dually annotated notes. Third, TraumaML is designed for annotation of EHRs for patients with diagnosis of PTSD and psychotic and mood disorders. It doesn’t cover all psychiatric diagnoses (e.g., addiction). However, the annotation schema and guideline can be incorporated, expanded, and modified to suit for different psychiatric conditions that extend beyond the scope of this project. Finally, the annotation guidelines were created with respect to the state-of-the-art in NLP and ML. This choice was reflected in the trade-offs of richness in clinical detail for support of modeling capabilities, particularly regarding the limitations in the size of reading frames and limitations in capabilities of few or one shot learning. Future models may implement new methods removing these limitations to create a gold standard assuming human parity.

## Conclusion

In this study we developed an annotation scheme and associated guidelines for marking indicators of trauma in psychiatric patient EHRs, undertook an annotation task with three domain expert clinicians, which has in turn, resulted in our creating a gold standard corpus for these indicators, the TEPC. Furthermore, we verified that the schema is learnable by ML algorithms by building a baseline model that will be improved in future work for use in analyzing large volumes of unlabeled clinical data to investigate the relationship between trauma (including types of events, perpetrators, and when the trauma occurred) and clinical outcomes.

## Figures and Tables

**Table 1:** Distribution of gold standard corpus.

	Count
Documents	101
Tokens	222,033
Span Tags	
**Symptom**	6,022
**Event**	800
**Substance**	604
**Temporal_Frame**	325
**Perpetrator**	237
Relation Tags	
**Grounded_To**	368
**Perpetrated_By**	271
**Sub-Event**	41

**Table 2:** Mean pairwise IAA for each label and attribute as measured using instance-level F1 and relaxed F1 for relation tag.

Label	Mean F1	Relaxed F1
**Event**	0.660	
**Perpetrator**	0.621	
**Substance**	0.715	
**Symptom**	0.706	
**Temporal_Frame**	0.460	
**Grounded_To**	0.599	0.941
**Perpetrated_By**	0.636	0.949
**Sub-Event**	0.367	0.500
**Childhood_Trauma**	0.917	
**Event_Type**	0.839	
**Negation**	0.984	
**Not_Current_Symptom**	0.881	
**Perpetrator_Type**	0.807	
**Temporal_Type**	0.792	

**Table 3:** Per-label results of span-level (A) and relation classifier (B) for the baseline model.

A. Span-Level model					
	precision	recall	f1-score	support	Human
**Event**	0.549	0.719	0.622	320	0.660
**Perpetrator**	0.192	0.500	0.278	50	0.621
**Substance**	0.476	0.555	0.513	346	0.715
**Symptom**	0.629	0.754	0.686	2,665	0.706
**Temporal_Frame**	0.264	0.494	0.344	89	0.460
**Macro Avg**	**0.422**	**0.604**	**0.489**	**3470**	

B. Relation Classifier				
	Precision	Recall	F1	Support
**Perpetrated_By**	0.732	0.703	0.717	101
**Grounded_To**	0.885	0.568	0.692	95
**Macro Avg**	**0.806**	**0.638**	**0.705**	**196**

## Data Availability

Deidentified data is available at the National Data Archive (grant reference R21MH125076–01). Code used for pre-annotation, error analysis, and baseline modeling can be found on our GitHub page <https://github.com/TraumaML>.
